# Complete Aphonia, Successfully Treated

**Published:** 1844-01

**Authors:** Martial Dupierris

**Affiliations:** Havana


October 20th, 1843.

---

Art. III.—*Complete Aphonia, successfully treated.* By Martial Dupierris, M.D., of Havana.

J. S. Rosh, aged twenty-six, of Alabama, visited Havana in January last, with complete aphonia.

He stated that in April preceding, he was taken with an acute pain in the left side, for which he was bled, blistered, &c., which relieved the acute pain, but he was troubled with a dull heavy pain, which, in despite of every remedy prescribed, continued until July, when it extended to the epigastric and right hypochondriac regions. His throat was also attacked about the same time, which soon resulted in complete aphonia; his eyes also became very weak and inflamed. He was then put upon the use of iodine, and a constant counter-irritation was kept up over the chest and epigastric region, by which he got some slight relief from the pains in the side, &c.; the throat, however, remained the same. For the cure of the latter, various gargles, the application of lunar-caustic, &c., were tried in vain. His physicians made every effort to cure him until December; they then advised him to visit Havana, and try the benefit of a milder air, as they discovered whenever the weather became damp and cold, his throat was more swelled both internally and externally, and more painful, with a considerable augmentation of the other pains of which he complained.

After being in Havana a short time and experiencing no relief, he placed himself in the hands of a physician who cauterized the throat internally, three times a week, with a saturated solution of lunar-caustic, and used blisters externally; under this treatment the pain and swelling of the throat was somewhat alleviated, but the loss of voice continued. After trying this treatment six weeks he placed himself under another physician. The latter, discovering considerable tenderness on pressure over the cervical and dorsal vertebrae, and a slight lateral curvature of the spine, with constant twitching of the muscles of the face, thought proper to apply issues to the spine, and prescribe constant rest in bed. The patient, besides, took occasionally carb. of iron, combined with sup. carb. soda. After the issue, which was opened over the middle dorsal vertebræ, had been kept discharging about a month, the pains experienced in the different parts of the body disappeared, save in the throat; his eyes, however, continued weak, there was no diminution in the twitching of the muscles of the face, and the aphonia continued. An issue was then opened on each side of the neck, just below the lobe of the ear; in a short time the twitching of the muscles of the face ceased; his eyes became less painful and sensitive; and his voice slightly restored so that he could speak in a low whisper. The issue over the spine was then allowed to heal after being kept open six weeks, and he was advised to use the sea-bath daily, which he continued to do for a month. At the end of this time his general health was much improved, there was much less tendency to swelling of the throat and no pain of the body, but the voice was still very feeble.

It was at this period he consulted me relative to the propriety of trying electricity. I advised him first to use a caustic of nitrate of mercury to his throat, and to take internally pills of nitrate of silver, which he agreed to; under the use of these remedies the voice daily became stronger, and in two weeks he could speak quite plainly and without pain. He then left Havana for Alabama, and has since written a letter of thanks to his last two attendants stating that his health and voice were entirely restored.

